# Characterizing opioid use disorder patients who have received medication trials with both buprenorphine and methadone

**DOI:** 10.1186/1940-0640-10-S1-A71

**Published:** 2015-02-20

**Authors:** Christine M Wilder, Paul Horn, Theresa Winhusen

**Affiliations:** 1 Department of Veterans Affairs Medical Center, Cincinnati, OH, 45220, USA; 2 Department of Psychiatry and Behavioral Neuroscience, University of Cincinnati College of Medicine, Cincinnati, OH, 45229, USA; 3 Department of Pediatrics, Cincinnati Children’s Hospital Medical Center, Cincinnati, OH, 45229, USA

## 

Since the approval of buprenorphine for treatment of opioid use disorder (OUD) in 2002, it has become increasingly likely that some individuals with OUD will have been treated with both buprenorphine and methadone at different points in time. However, this emergent group of patients has not been well described. We completed a retrospective cohort study of individuals at the Cincinnati Veterans Administration with treatment episodes for both buprenorphine and methadone and compared this group with individuals who received treatment with buprenorphine only or methadone only. Between January 1, 2006, and May 1, 2014, 163 veterans had both buprenorphine and methadone treatment episodes for OUD. We extracted information from the local administrative and pharmacy databases to describe these individuals. Individuals with treatment episodes for both medications had significantly higher levels of comorbidity with other substance use disorders (specifically, alcohol, benzodiazepine, cannabis, and cocaine use disorders) as well as mood and anxiety disorders than those who received either buprenorphine or methadone only. They also used a disproportionate amount of urgent and emergency services compared to individuals prescribed either buprenorphine or methadone only (mean of 17.8 billable days versus 11.3 for buprenorphine only and 10.4 for methadone only, p < 0.0001). We conclude that these patients represent a treatment-resistant group that would benefit from earlier identification and more intensive intervention.

